# Integrated pond aquaculture and regional identity: ethnobiology of the golden humped tench of Poirino highlands, Northwest Italy

**DOI:** 10.1186/s13002-022-00529-5

**Published:** 2022-04-11

**Authors:** Alessandro Delpero, Gabriele Volpato

**Affiliations:** University of Gastronomic Sciences, Piazza Vittorio Emanuele 9, 12042 Pollenzo, Bra, CN Italy

**Keywords:** Ethnoichthyology, *Tinca Gobba Dorata*, Ethnoecological knowledge, *Peschiera*, Sustainable fish production, Gastronomic heritage

## Abstract

**Background:**

Social–ecological systems are based on particular species and on their direct and human-mediated interactions. The ‘golden humped tench’ or *tinca gobba dorata*, a variety of tench—*Tinca tinca* (L., 1758)—traditionally bred in artificial ponds called *peschiere* in Poirino highlands, northwest Italy, is one of such species. The aim of the study is to investigate the traditional farming of the golden humped tench, the associated knowledge, practices, and gastronomy, and to discuss the changes that the tench, the ponds, and their role in the local social–ecological system are going through.

**Methods:**

The data analyzed were collected in different locations of Poirino highlands during May–September 2021. Fieldwork included semi-structured interviews (*n* = 23) with current and former tench farmers about the breeding and gastronomy of the tench and the management of the *peschiere*. The interviewees’ selection occurred through an exponential non-discriminative snowball sampling, and interview transcripts were qualitatively analyzed through inductive thematic content analysis.

**Results:**

The golden humped tench has been farmed for centuries in ponds used also to water livestock and to irrigate cultivated fields, and managed by every peasant household in the area. This integrated aquaculture system is underpinned by detailed knowledge on the *peschiera* ecosystem and on the tench life cycle and supports a gastronomic knowledge that is part of the local heritage. The ongoing process of gastronomic valorization of the tench is sustaining the role of the fish in locals’ livelihoods and as a marker of regional identity, but it is also transforming tench farming, already threatened by livelihood change, pesticides, and invasive species, in controversial ways.

**Conclusions:**

We argue that ponds and tenches are core elements of the local social–ecological system, defining the cultural landscape and engendering a form of regional identity around them. Studying integrated aquaculture systems and associated knowledge and practices is relevant to design sustainable systems of food production and to address possibilities of conservation of biodiversity and livelihoods in aquatic environments.

## Background

Social–ecological systems are complex and integrated systems in which humans and nature coevolve through intimate and continuous relations [1, 2]. These systems are based on specific sets of (wild and domesticated) species and on their direct and human-mediated interactions [3, 4]. The relationships between humans and these other species are complex, deeply entangled, and rooted in specific ecologies and social contexts [5–7]. Ethnobiological investigations are in a great position to investigate such species and the systems in which they are embedded. Such investigations, when dealing with food species and associated knowledge, can further be framed within the theoretical contours of gastronomic ethnobiology [8]. Two directions in which ethnobiology has recently branched are the ones of aquatic ethnobiology (i.e., the study of the evolving interrelationship between people and aquatic organisms [9]) and ethnoichthyology (i.e., the study of the relations between people and fish and other underwater animals [10–12]). Indeed, fisheries in all their forms are integral to the social–ecological systems and livelihoods of local communities [13, 14], and fish farmers and fishermen hold a deep knowledge of the environments and species they interact with [15–17]. The livelihoods and identities of countless peoples are intertwined with the aquatic species they depend upon, within complex systems of management and use, informed by traditional ecological knowledge of these species and the ecosystems they inhabit [13, 18].

Drawing on the social–ecological systems approach, in this study we investigate the ethnobiology of the ‘golden humped tench’ (also known with its Italian name as *tinca gobba dorata*), a variety of tench—*Tinca tinca* (L., 1758)—traditionally bred in artificial ponds called *peschiere* (sing. *peschiera*) in the *Pianalto di Poirino* (Poirino highlands), northwest Italy. We merge this approach with an ethnoecological investigation of the traditional management of the ponds where tenches are bred, thus contributing to the study of freshwater fish–humans relations and embracing the call by Svanberg and Locker [10] to widen ethnobiological research in Europe to include fish and associated knowledge and management. This is indeed an urgent task because much ethnoichthyological knowledge is being lost [13], and the study of traditional knowledge and practices is relevant for the present and future capacity to produce food and to improve conservation and management strategies [19, 20].

The farming of the golden humped tench is a little-known traditional system of integrated aquaculture. Prein describes such systems as constituted by ‘sequential linkages between two or more human activity systems (one or more of which is aquaculture), directly on-site, or indirectly through off-site needs and opportunities, or both’ [21] (p. 128). An essential characteristic of integrated systems is that nutrients circulate supporting different livelihood strategies. In China, carps have been farmed in rice fields for thousands of years, in a system in which they eat rice pests and fertilize the plants, and the rice provides shadow and regulates water temperature, in an example of mutual relation at the core of peasants’ food systems [22]. In the delta aquaculture systems of the Mekong River, in Vietnam, pigs fertilize the soil, promoting the growth of rice and of microorganisms that become food for catfish and tilapia. The fish, in turn, feed from mosquito larvae as well as from rice pests, and rice plants provide shadow and filter toxins. While most studies on traditional integrated aquaculture focus on Asia [21], such freshwater systems are present globally in different forms [23]. Integrated aquaculture is increasingly regarded as a potentially sustainable form of food production to be approached systemically, in recognition of the fact that it includes several variables interrelating in a complex adaptive system [24]. These systems are knowledge-intensive [10, 21] and as such are a fertile ground of investigation from an ethnobiological perspective.

In this study, we frame the tench integrated aquaculture and associated knowledge and practices as tools for sustainable food production and elements of a collective cultural identity rooted in the region of Poirino highlands. Such regional identity is a kind of spatial identity referring to a feeling of belonging to an area at the mesoscale and grounded in regional history, language or dialect, livelihoods, gastronomy, and their elements [25–27]. Regional identities are premised on places as emotional bounded areas [28], as well as on species and foods as core elements of these emotional ties. Such identities refer to communities identifying themselves with the material and non-material manifestations of the place-based coevolutionary process underpinning social–ecological systems.

In light of this, the aim of the study is to investigate the traditional farming of the golden humped tench in Poirino highlands, the associated knowledge, practices, and gastronomy, and to present and discuss the changes that the tench, the ponds, and their roles in the local social–ecological system are going through. Through ethnobiological fieldwork, we explore the knowledge and practices around tench and about the management of the multi-use ponds where tenches live, discussing them as tools for sustainable food production and as elements of a regional identity with tenches and ponds at its core.

## The tench in Poirino

The tench is a medium-sized freshwater cyprinid native to Asia and Europe and now widespread globally, being regarded by some scholars as a globally invasive species [29, 30]. Typically found in calm, shallow, muddy, and vegetated waters in lakes, rivers, and ponds, the tench is a generalist predator of macroinvertebrates and zooplankton, including insects, mollusks, and aquatic vegetation, tolerant of low levels of oxygen, and thus thriving in benthic areas of eutrophic waters [31, 32]. It is active at night, spending the days below the macrophytes and benthic substrates [33].

**Fig. 1:**
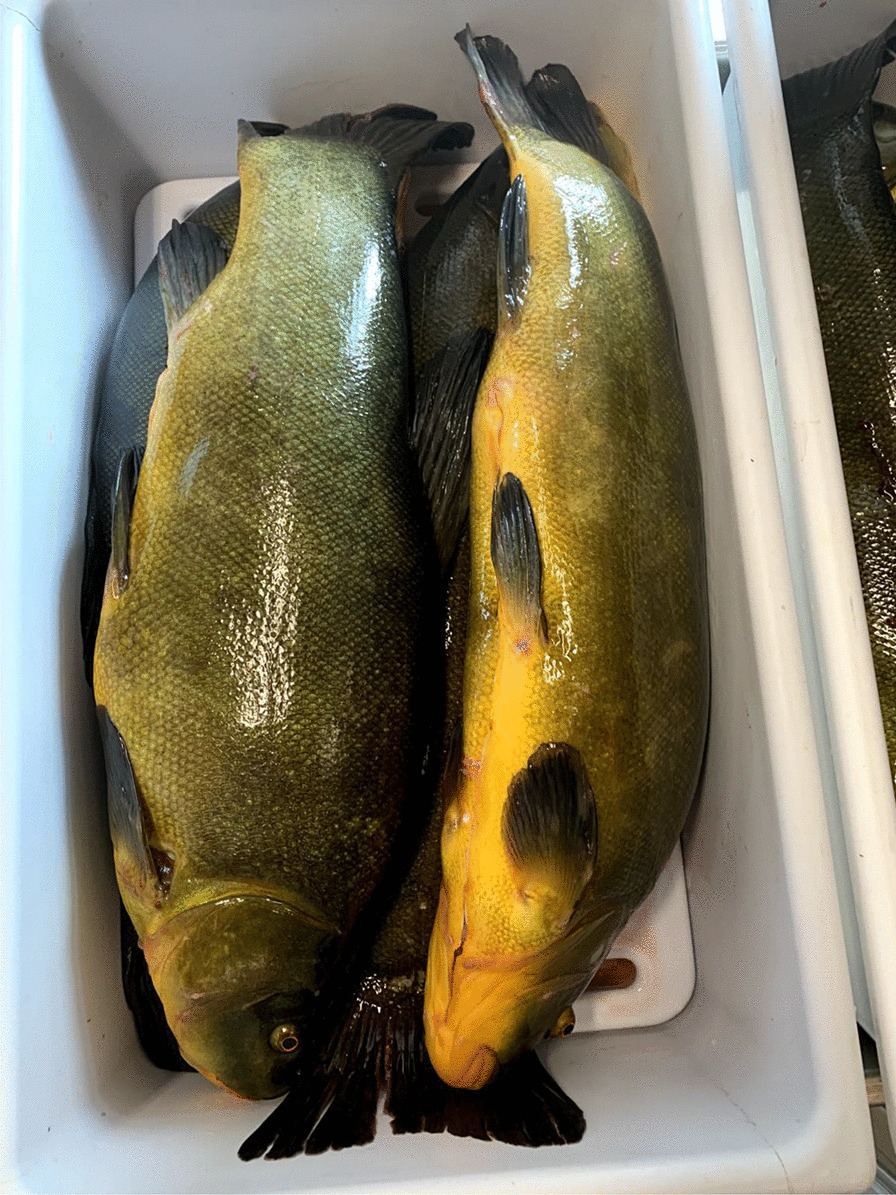
Adult golden humped tenches (Alessandro Delpero)

The tench is an important gastronomic and angling freshwater fish, traditionally farmed in many of its native areas. In Italy, it is widely distributed in rivers and lakes and historically farmed in systems of integrated aquaculture (e.g., with rice and carps in the rice fields of Vercelli, northwest Italy). It is also bred in the hundreds of artificial ponds that characterize the Poirino highlands, where the tench has been for centuries a constitutive element of the local social–ecological system [34]. The golden humped tench is named after its dorsal gibbosity and a skin that is graphite gray or green on the back and goldish on the sides (Fig. 1). In the ponds, tenches live deep in the water column, on the bottom during summers and winters, which they spend in a largely inactive state. Sexual maturity is reached between the third and the fourth year. Batch spawning occurs in shallow, densely vegetated waters from May to August, the adhesive eggs being broadcasted over vegetation close to the border of the pond, where water is shallower and where they receive enough heat from the sun. Eggs hatch after about 3 days, larvae stay attached to aquatic plants. Fries feed from zooplankton, crustaceans, and insect eggs [35].

Tenches have been farmed in Poirino for centuries [36]. Tench farming constituted a key livelihood activity for local peasants, as the tench has been an important food and protein source across the year and especially during winters, and an important item of exchange, gift, and trade. The history of the *peschiere* and tench farming goes back at least to the *Statuti di Bra* of 1461 (in turn ascending to the twelfth century), which fined the thieves of fish from the *peschiere*, to a reference to tenches being used to pay taxes from the thirteenth century, and to a mention in a document of 1577 to the water of a *peschiera* used to power a mill [36, 37]. There were hundreds of *peschiere* and a flourishing tench production in Poirino for the following centuries, and at the beginning of the twentieth-century golden humped tenches were sold in Turin and other cities. Tench farming was a rather seasonal (spring to autumn) livelihood activity, integrated with cattle husbandry, agriculture, and weaving in a loom for the textile factories of the nearby town of Chieri (until the crisis of the sector in the 1950s). Since the 1960s, tench farming in general has declined drastically, due to wider socioeconomic changes such as outmigration from rural areas and urbanization, and detachment of livelihoods from the primary sector with industrialization. If until the Second World War every family had a *peschiera*, since then the number of *peschiere* and the role and relevance of the tench (as a proxy of the role and relevance of the Pianalto area in the locals’ livelihoods) have decreased. In spite of this decline, golden humped tench aquaculture is still practiced among those families who maintained a *peschiera* active and managed, across generations, until today. As we shall discuss, the tench has also caught renewed interest as a traditional and typical food, being promoted and adopted as a sustainable form of aquaculture and sustaining a regional identity rooted in the local gastronomic heritage.

## Materials and methods

### Study area

The *Pianalto di Poirino* (‘pianalto’ means ‘upland’ or ‘plateau’ in Italian), where the golden humped tench is farmed, includes 24 municipalities in the provinces of Asti, Cuneo, and Turin in the Piedmont region, northwest Italy. A *pianalto* is a moraine geological formation typical of the Italian Pre-Alps and of the high Po Valley and formed by deposits left by glaciers moving south from the Alps during the last glacial period. The Poirino highlands cover about 400 square kilometers of hilly and irregular terrain between the Turin hills to the north and the Roero hills to the south. It has a north–south axis of some 40 km and an east–west axis of some 15 km, an area well defined geographically by the escarpments delimiting it, with a slight slope toward the Langhe hills to the south and the Po valley to the east.

The region is characterized by about 700 mm of rain per year, with dry summers, and a continental and sub-arid climate. The absence of rivers and streams in the uplands, due to the area being geographically cut off from the surface water flowing down the Alps, compounds with the climate to generate a water deficit during the hot summers. Geologically, the highlands are outlined by alluvial deposits, especially silty clay alluvial deposits. The region is defined by red soils, rich in iron and clay, which are ideal for the production of bricks. These soils also hold very well surface waters, feature that underpinned the emergence of tench farming in the area. The economy of the Pianalto is mainly based on agriculture, the main produce being cereals, vegetables, strawberries, asparagus, and tench. Some textile industries have been active in the area since the eighteenth century, and there still are some draperies, as well as, in connection with the clay soils, a small-scale industry of brick making.

### Field research

Fieldwork was carried out in May–September 2021 in different locations of Poirino highlands, gravitating around the three main towns of Poirino, Pralormo, and Ceresole d’Alba. We conducted in-depth (60–90 min) interviews with 23 former and current tench farmers (16 men and 7 women). We used anthropological fieldwork methods such as semi-structured interviews, focus groups, and participant observation as tools for collecting data [38]. Semi-structured interviews were constructed to gather diachronic and synchronic ethnoecological and ethnoichthyological information about *peschiere* and tench farming, with the goal of eliciting qualitative information, experiences, and perspectives about this livelihood practice. Two focus groups, one in Poirino and the other in Pralormo, were conducted with current and past tench farmers to explore the traditions, changes, and challenges related to tench farming in the Pianalto. To elicit information on the ethnoecology of ponds and on the ethnobiology of the golden humped tench, interviews and focus groups explored the history, characteristics, and management of the ponds; tench breeding and its gastronomic uses; social and cultural value of the tench; contemporary challenges to the maintenance of the *peschiere* and to the continuity of tench farming; and the contemporary use and value of the golden humped tench in light of the undergoing initiatives for its valorization as a typical product. The information gathered through interviews and focus groups largely overlapped in content, albeit focus groups tended to converge more on the relations between stakeholders in tench farming and marketing, and on the contemporary threats to the continuation of the practice. Participant observation included joining tench fishing in ponds and tench cooking with four interviewees.

The interviewees’ selection occurred through an exponential non-discriminative snowball sampling [39] once the first participants, who were acquaintances of the first author who resides in the area, had been identified. The criteria used to select the interviewees during the snowballing process were limited to past or present experience in tench farming, processing, and cooking, as well as in the ponds’ management. We continued snowball recruitment until we considered to have covered the widest possible constellation of actors in terms of age and experience in tench farming, farm size, and degree of market engagement.

Participants were given an explanation of the methodology and aims of the study, and informed consent was obtained verbally prior to the interviews. Throughout the field study, the American Anthropological Association ethical guidelines [40] were followed. All the interviewees’ responses in the study are anonymized. Interviews were recorded and transcribed, transcripts were qualitatively analyzed through inductive thematic content analysis, and codes, concepts, and categories were generated during analysis [41, 42]. Some ten categories were obtained from the analysis, including: tench management; tench gastronomy; pond management; historical aspects; systemic links; market engagement and valorization; change in tench farming; threats to the system; cultural identity around tenches and ponds; tenches, ponds, and social life.

## Results and discussion

Below, we present the results from fieldwork in relation to the ethnoecology of the *peschiere* and the ethnobiology of the golden humped tench. We first describe the *peschiere*, their management, and their key role in the Poirino highlands’ integrated aquaculture system and then address the breeding, gastronomy, and social and cultural role of the tench among locals. While tench farming has lost importance in local peasants’ livelihoods, the number of ponds has decreased, and new challenges threaten the continuity of the practice, a process of valorization of the fish as a typical gastronomic product is emerging. We discuss these results arguing for the relevance of studying traditional integrated aquaculture systems as sustainable forms of food production, and for the important role of the tench and the ponds for a collective regional identity in Poirino highlands.

### Peschiere

**Fig. 2:**
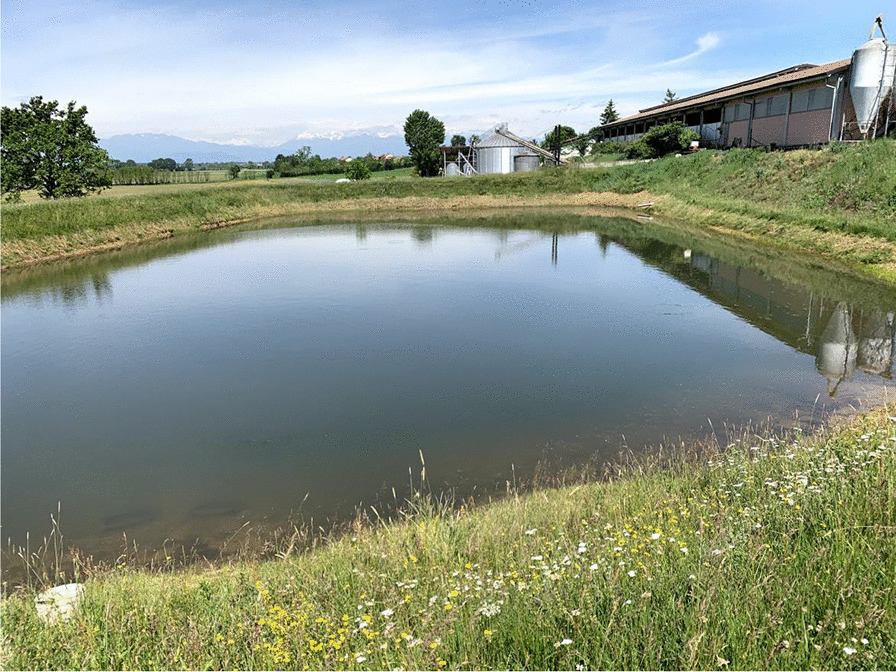
A *peschiera* near Pralormo (Alessandro Delpero)

The defining element of the Poirino highlands is hundreds of *peschiere*, i.e., artificial ponds dug across centuries as watershed basins and used as water reservoir (especially during dry summers), for irrigation, livestock watering (i.e., cows, chickens, geese, horses, and/or sheep), and tench farming (Fig. 2). In the long-lasting coevolution between the highlands and their human inhabitants, *peschiere* were born as holes left by the extraction of clay to make bricks to build houses. *Peschiere* were dug in chosen places, particularly in red clay soils, and the ground removed was used to build the banks. Often, one bank was built less steep to allow livestock to enter the pond. Red clay is indeed the defining material of the Pianalto, the one that underpins the same existence of tench farming there. ‘The clay from our soils is so hard and resistant that is ideal for bricks. The oldest farmhouses in the area have been built with that kind of brick,’ an interviewee states. Ponds and furnaces to cook the bricks were intertwined, and tench farming stems from house building and brick making. Over time, thanks to their positioning in relation to the fields’ slopes, these holes get filled with rainwater and surface water, becoming ponds. The ponds owned (or previously owned) by the interviewees have a variable shape (e.g., rectangular, circular, triangular), and their surface area varies between half a hectare and two hectares. The average depth is about one meter and a half, with a maximum, in the center, of some three meters. The interviewees explained that *peschiere* must be deep enough to hold water for irrigation, but not so deep as to compromise the farming of tenches there.

Ponds in Poirino highlands constitute important wetland ecosystems [36]. Each *peschiera* has its own ecological and biological characteristics in accordance with location, size, and management. The interviewees stressed that ‘they are teeming with life,’ referring to the biodiversity in the ponds. The water, fertilized by livestock entering the pond to water (or to cool during the summer), and thus rich in organic material, sustains a whole ecosystem of micro- and macro-organisms (e.g., bacteria, aquatic plants, insects, frogs, tenches). Livestock fertilization induces the growth of a complex feeding substrate, rich in microorganisms and invertebrates, on the bottom of the pond [35]. Indeed, interviewees report the presence there of a slush called *nita*, which is also used as fertilizer. They refer to the water as ‘black, very dirty, at time so dark that you could not see the fish,’ as noted by one interviewee. Those darkness and dirtiness are due to the abundant suspended organic material, which is the substrate for the whole *peschiera* ecosystem structured in a complex trophic web. The interviewees report that the amount of organic material entering the *peschiera* through livestock must be balanced (in accordance with each pond’s size, depth, and biological condition) for tenches to thrive. Livestock activities in the ponds are thus regulated based on the requirements of tenches as well as to those of livestock. As such, *peschiere* provide food with a non-intensive human management, the ecosystem regenerates starting with livestock activities and ecologically transforming the organic material deposited in the ponds into tenches.

The *peschiere* were central to livelihoods within the social–ecological system and peasant economy of the Poirino highlands. Ponds were important for irrigation, clay extraction, and livestock watering, but they were also social places. The *peschiera* used to be the epicenter of summer activities and social life. Some folk songs collected by Lusso [43] evoke sultry summer days around the ponds, swimming, trying to catch a tench or two, or chasing frogs. This material and cultural importance of the *peschiere*, and their concurrent ecosystem dynamics, illustrate the intimate and coevolving relation between humans and the place they inhabit. The Poirino highlands and their *peschiere* manifest here as cultural landscapes [44, 45], as ‘patterns that cultures imprint on the land’ [46] (p. 3081).

### Management and use of the golden humped tench

Farming of the golden humped tench in *peschiere* began when tenches, caught for long time in rivers of the region, were transferred from the Po or Tanaro rivers or from local streams (Banna, Rioverde, and Ricchiardo) to the ponds in the highlands of Poirino. Once an upland population of tenches living in ponds was established, sourcing from rivers became rarer as ponds were replenished through reproduction and through gifts and exchanges of fries among households. This exchange was crucial for the reproduction of the system and for the maintenance of the golden humped tench populations and their characteristics.

The management of the fries for ponds’ repopulation emerges from the interviews as a key practice of the system. *Peschiere* are heterogeneous in their management; some, usually the bigger ones, host a full tench population reproducing year after year, while in others (the smaller) fries are reintroduced every year in spring (‘about a thousand fries for an average pond’), after the tenches of preferred size (80–100 g or a span in length) have been caught the previous autumn (tenches take about 2 years to reach the right size for traditional processing methods). This heterogeneity points to a breeding system coexisting with a ‘fattening’ system, whereby the former provides fries to the latter through gifts, exchange, and sale. The interviewees stress the importance of controlling tench populations in the *peschiera* to avoid overcrowding, in which case the tenches would grow smaller and darker, and of fishing only tenches of the right size, the others (e.g., those too small and reproductors older than 3 years) being released back in the pond. Overcrowding is inferred partly by direct observation of the tenches and partly by the overall small size of the fish, which indicates overcompetition in the pond. According to the interviewees, in autumn the fish should be of a certain size, and if this is not the case than one of the causes could be overcrowding (which could be confirmed or not by fishing trials), in which case they would fish the tench surplus out of the ponds. The biggest and oldest tenches are six to seven years old and can reach one kilogram and a half of weight, time when they do not reproduce anymore and are thus consumed. The interviewees recognize these aging individuals by the fact that ‘females carry few eggs and their bellies are not swollen, and they don’t move much anymore.’ The consumption of these older tenches is an important, symbolic occasion for the family and takes rather place on Sundays.

**Fig. 3:**
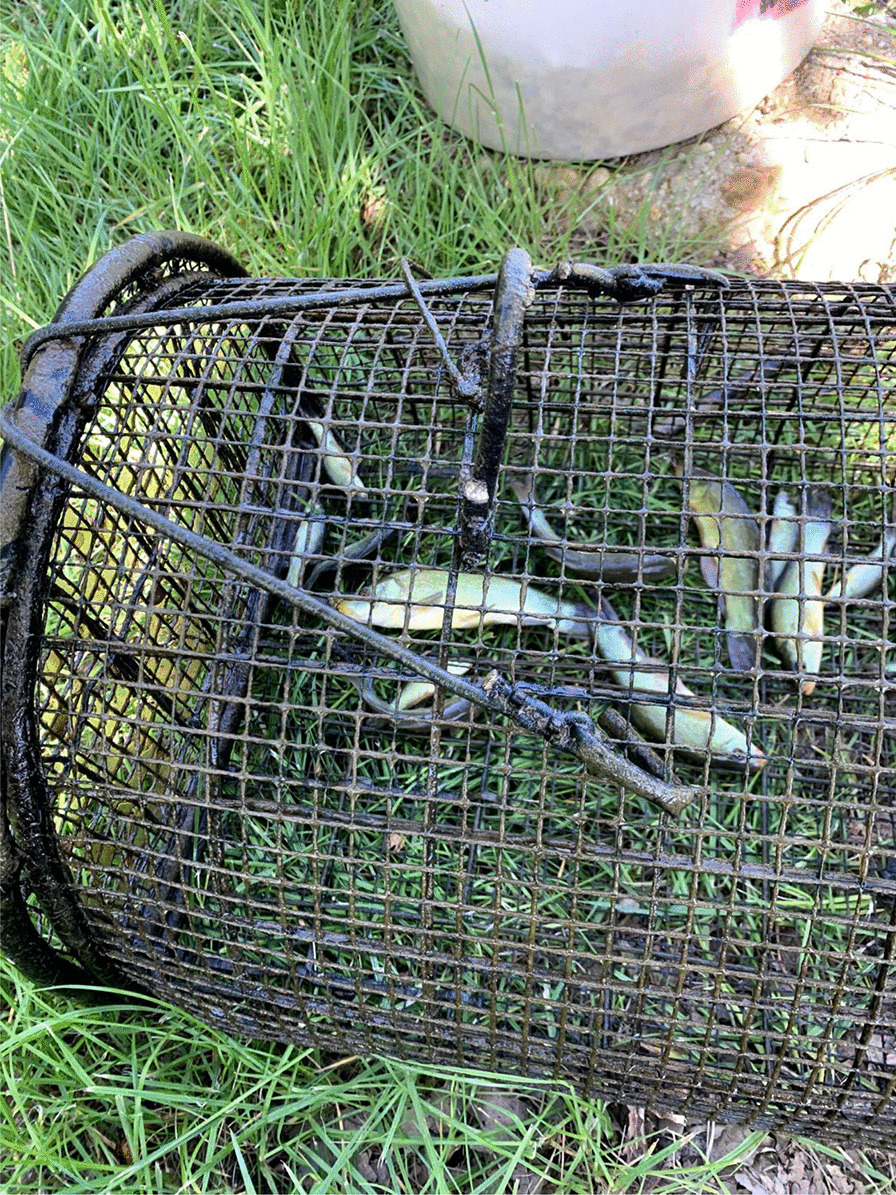
Fish trap with golden humped tenches (Alessandro Delpero)

**Fig. 4:**
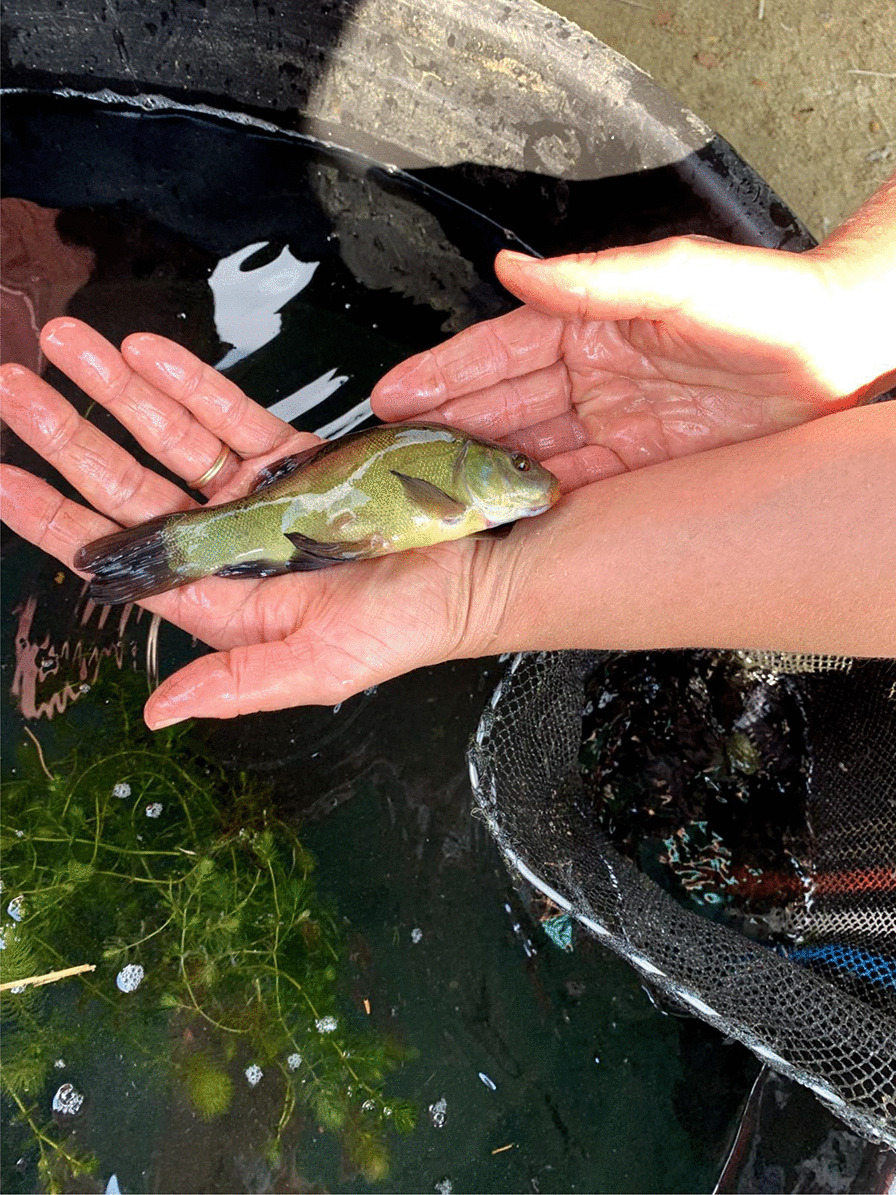
Selecting tenches caught in the fish trap (Alessandro Delpero)

Fishing tenches out of a *peschiera* is an important material and cultural practice, subjected to collective rules and norms. Fishing rules have long been present in the area, regulating human–tench relations within a complex and dynamic system. At the beginning of the twentieth century, there were rules about minimal size and a ban on fishing in June, month in which spawning occurs. The fishing season goes from March to October. Fries, to be sold or gifted, are caught with the so-called *trübia*, a tight mesh net. Fishing tools for adult tenches include the *bertavel* (cylinder-shaped lobster pots or fish traps made with common osier, using a piece of bread, dry polenta, food remaining, eggs’ shells as bait, ‘almost anything would do’) (Figs. 3, 4). ‘Like pigs, tenches eat all the food scraps of the house’ according to one interviewee. Fish traps are placed in the pond during the evening and collected in the morning, because tenches move and feed at night. Another tool is the *tondin*, a net tied between two flexible sticks, introduced in the pond for some minutes and then lifted, in an attempt to catch fish that would have moved above the net in the meantime. The *rabast* (literally broom), a bottom trawling small net, instead, is used during summer, when the tenches tend to stay on the bottom and enter periods of a summer lethargy, ‘with the head in the slush.’

**Fig. 5:**
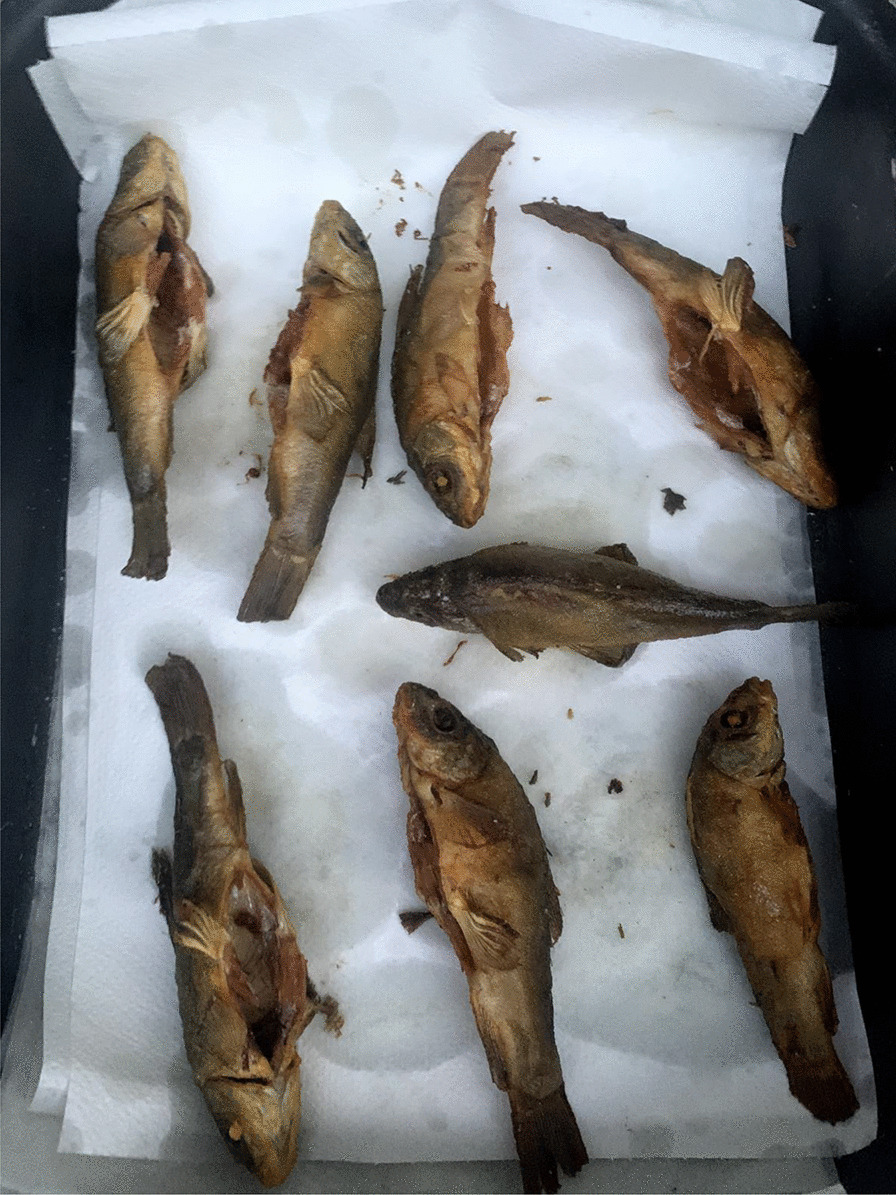
Fried tenches (Alessandro Delpero)

Tenches are taken home alive, as ‘they must be processed soon after death or produce foul smells.’ They are eviscerated and washed, and then, crucially according to the interviewees, they are wrapped around a dish cloth and left in a cool place overnight. This is regarded as an important step to harden the fish, which would otherwise flake off in boiling oil. ‘It is a knowledge that every local has; many have stopped eating tench because they don’t know how to prepare it,’ claims one interviewee. Tenches are then fried and they can be eaten all, including head, bones, and tail (Fig. 5). Tradition wants the fried tench to be bitten first from its hump. The meat is appreciated for the delicate flavor. A traditional way of processing the tench, and fish more in general in northwest Italy, is called *il carpione*.^1^ Tenches are first fried and then marinated in small casks containing an emulsion obtained from a *soffritto* of garlic, onion, sage, vinegar, and water. Tenches *al carpione* are prepared especially in autumn, as a yearly ritual, to preserve them and be consumed during the winter (while today *il carpione* is kept in the fridge, a cellar was traditionally used). Tenches were taken out of the cask and eaten to be fully consumed by the end of the winter, when the fish would become too strong and sour. Tenches *al carpione* were thus a crucial protein source during winters, in a historical peasant context in which meat was seldom consumed.

Tenches are also described as important means of exchange. They are gifted to feed social relations and reciprocity, as well as sold in markets. One of the interviewee reported that the father used to rely on some workers during the harvest of asparagus and zucchini and that the payment consisted in the agreed cash plus one or two tenches. The exchange and gift of fries and tenches among the inhabitants of Poirino highlands is an important practice that reproduces the system and tightens the community, rooting a collective identity into the region and its gastronomic manifestations. The continuous exchange and movement of fries between ponds may also underlie the high level of genetic diversity among golden humped tenches and within ponds as reported by Presti et al. [47].

### Loss, recovery, and valorization

With industrialization and urbanization in north Italy after the Second World War, *peschiere* lost their role in local livelihoods and food system [43]. Many families abandoned backyard and small-scale livestock husbandry, and *peschiere* also decreased in number. Some have been buried to make room for buildings and infrastructure, and some others have been abandoned and on their way to dry up [37]. In Ceresole d’Alba, the primary school has been built on the former so-called *wide pond*, once used as water collection site and as ‘tench reservoir’ given the high numbers of tenches in there. The *long pond* of Ceresole has instead been replaced with recreational facilities [43].

Although it has witnessed a decline during the last century, the farming of the golden humped tench continues and is gaining attention with its qualification as traditional and typical product [48]. Several tench farmers maintain a small-scale production geared toward home consumption, gift-giving, and informal sales, and there are few commercially oriented farmers too. Among the initiatives undertaken to valorize the golden humped tench as a niche gastronomic product, there are the constitution of a Slow Food presidium^2^ among the producers and obtaining the Protected Designation of Origin (PDO) designation.^3^ Today, the Associazione dei Produttori della Tinca Gobba Dorata del Pianalto di Poirino^4^ is involved in improving and valorizing the tench of the Pianalto and in maintaining and restructuring the *peschiere*.

Two emerging trajectories are detectable in relation to the maintenance and valorization of tench farming and gastronomy in the Pianalto. In Pralormo, there are several households managing *peschiere* and making tench farming a complementary economic activity to cash cropping and livestock husbandry. The tench seems there to retain much of its traditional social and cultural role, and interviewees report that, in season, every weekend people gather in private houses or communal places (e.g., the ProLoco organization) to eat fried tenches and frogs in a convivial setting. Every year, from beginning of spring through summer, the town’s inhabitants meet to eat fried tench, and this convivial consumption reproduces the role of the tench in local culture and sociality. It is the food offered to Sunday guests and in family meals. In the hamlet of Crivelle, locals eat *tinche al carpione* every day of a specific week of July, celebrating the end of the tench season. Tenches stand for childhood memories, for summer days, and long-gone friends. This conviviality informs a regional identity rooted in a gastronomic heritage that has tench at its core. As argued by Svanberg [14] for fermented fish in the Faroe Islands, tench in Poirino highlands is not only a food source, ‘but its consumption is also an important act of identification and solidarity’ with the local culture and territory.

Ceresole d’Alba, a town south of Pralormo, is witnessing a second trajectory geared toward the patrimonialization of the golden humped tench through its involvement into the Slow Food Presidia’s project. In Ceresole, there has been since the 1990s a recovery and a reconfiguration of tench farming for the market, and new *peschiere* have been built by producers who have also intensified the form of production (e.g., using cereals as feed). Tench gastronomy has been adapted too (e.g., smoked tench, *tinca al carpione* in glass jars), its products made available all year, and these niche products are now sold in the national market. An overall trend of wider engagement by tench farmers with the market is noticeable across the Poirino highlands, hinting at a commodification of tench farming whereby tenches are increasingly marketed as typical products. Buyers include local restaurants (in connection with gastronomic tourism in the region), privates, and quality and typical products’ shops.

However, interviewees report that tenches are most often sold informally, and many did not join the DOP or Slow Food certification systems, as these require providing feed to the tenches and keeping livestock out of the ponds. Similar problems arise from the European norms of hygiene and food safety. The adaptation of many small-scale producers (e.g., of mountain butter, of raw milk cheese) to these norms has been difficult at least, and they have a hard time following disciplinaries and protocols, preferring to stay out of them [49–51]. The norms, in reference to tench farming, ban the direct fertilization of the ponds by livestock, as no livestock is admitted to water in the pond due to hygienic reasons; instead, only manual fertilization is allowed. Furthermore, to prevent animals or people from falling in the ponds, the rules demand ponds to be fenced, which implies a breaking of the link between livestock and ponds [50]. Interviewees have repeatedly voiced their concern about the impact that the application of these norms has on tench farming, pointing to the livestock–pond link as essential in the tench farming system. They also claim that the breaking of this critical link reflects all the way down to the taste of the tench in the kitchen table.

In order to promote and valorize local and traditional foods, several towns and localities are organizing gastrofestivals and gastronomic fairs [52, 53]. Tench and asparagus are important symbols for the Pianoro inhabitants, the core of local gastronomic heritage. Fairs where locals proudly display and sell fries for pond repopulation, reproductors, and tenches to consume have taken place for centuries, and these fairs renew yearly the link between people, food, and the place and convey a regional identity expressed in foods. Since 1954 until the 2020 COVID-19 Italian lockdown, a fair of tench and asparagus took place in Poirino every second Sunday of May. Local restaurants and associations used to sell fried tenches and other local produce and dishes. Several interviewees stressed the historical roots of the fair, recalling a time when, in years with abundance of tenches in the ponds, ‘every Sunday of May and June was like a fair, everybody joining to fry the fish from the ponds.’

### The Anthropocene in the pond

According to the interviewees, the recovery of tench farming and valorization of the golden humped tench are accompanied by new threats and challenges. The reproduction of the role of the tench within the local social–ecological system is being compromised by a number of factors, which reflect wider socioeconomic and environmental changes (e.g., use of pesticides, invasive species) impinging on the *peschiere*. Interviewees report that pond ecosystems have been affected by the intense use of pesticides and synthetic fertilizers in the cultivated fields of the Pianoro. These practices represent a source of pollution of the waters of the ponds. Tenches, feeding from the bottom, are affected by insecticides and herbicides that accumulate in the sediment [54]. Older interviewees compared the *peschiera* ecosystem through time, as they spent their childhood playing around the pond and observing its life: ‘Time ago, our ponds were full of life! Now, with pesticides used in the fields around, little is left.’ Pesticides have here poisoned a set of life-supporting mechanisms, i.e., those associated with the flow of the water between the fields and the pond and their cross-fertilization.

**Fig. 6:**
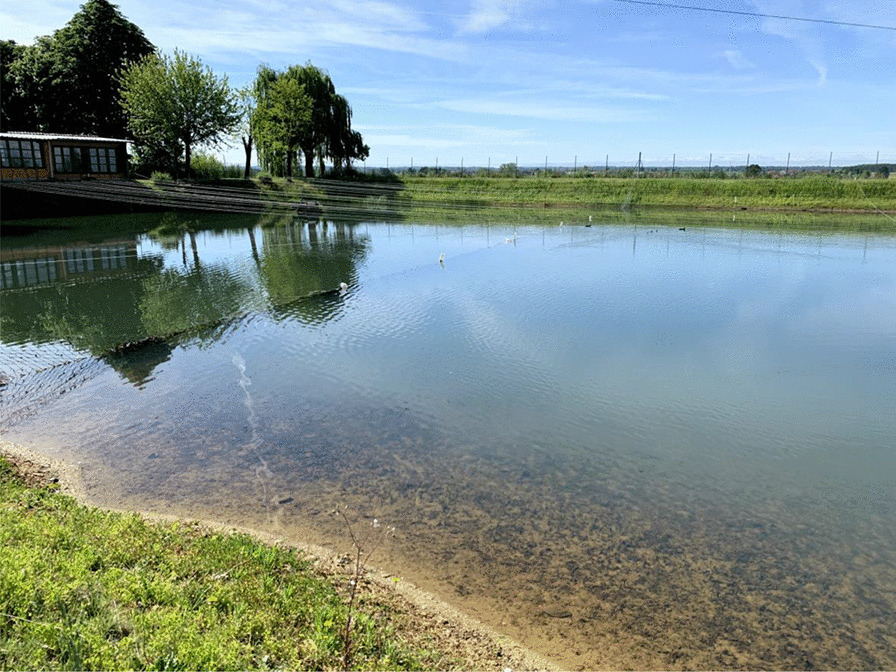
*Peschiera* with a system of crossing lines to prevent cormorants’ predation on tenches (Alessandro Delpero)

**Fig. 7:**
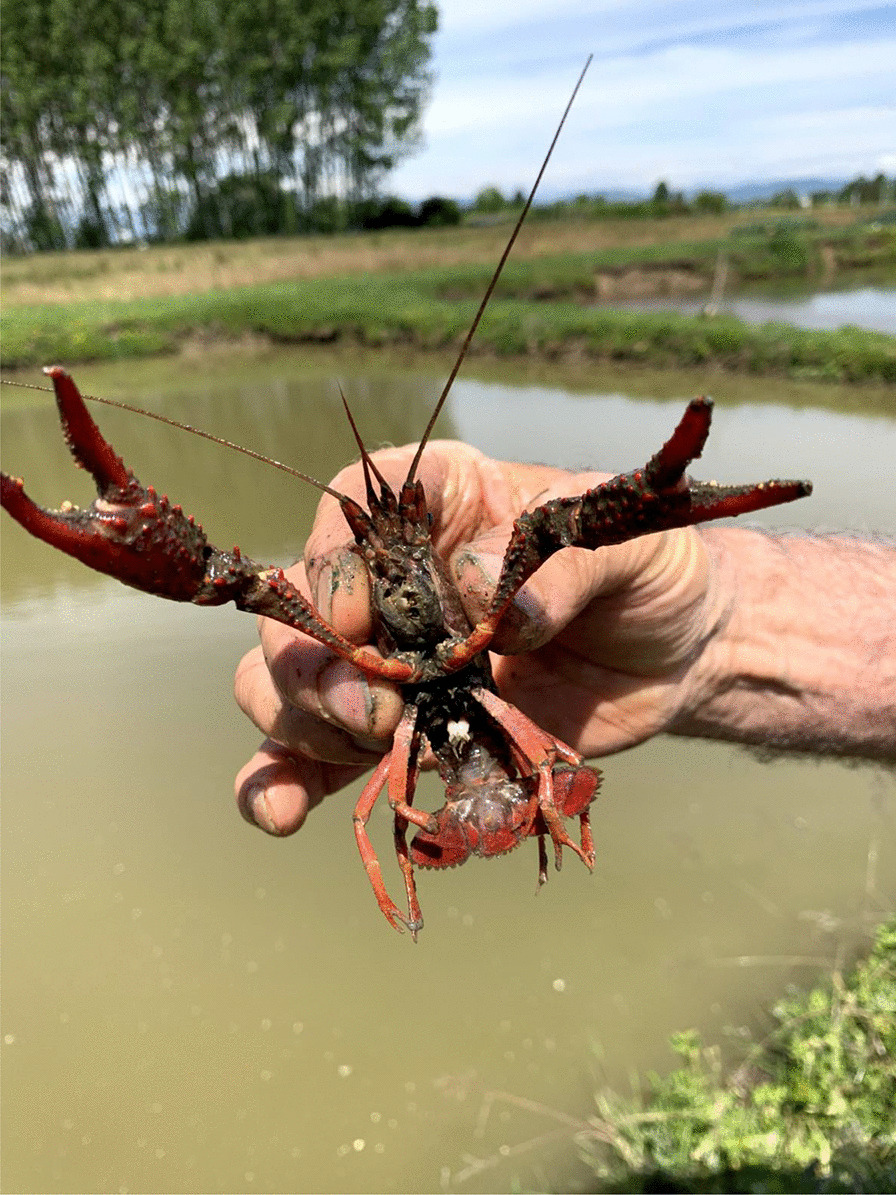
A Louisiana crayfish from a *peschiera* (Alessandro Delpero)

Another widely reported threat is the increasing number of alien predators and competitors, i.e., species that were introduced or that expanded their range to the Poirino highlands (e.g., due to climate change). The fauna of Italian rivers, lakes, and waterways has witnessed a high number of biological invasions in recent decades [55, 56]. Among these invaders, the interviewees mentioned cormorants, i.e., *Phalacrocorax carbo* (L., 1758), American bullfrogs, i.e., *Lithobates catesbeianus* (Shaw, 1802), Louisiana crayfish, i.e., *Procambarus clarkii* (Girard, 1852), and aquatic turtles (probably pet turtles released in the highlands’ waterways in recent years), which consume eggs, fries, and adult tenches. Nutrias, i.e., *Myocastor coypus* (Molina, 1782), frequent the ponds and damage their banks by burrowing, while swan mussels, i.e., *Anodonta cygnea* (L., 1758), change the characteristics of the pond ecosystem. Cormorants are voracious fish eaters that have in recent decades expanded in range and numbers into inland European waters [57]. ‘One or two cormorants can heavily affect tench populations, emptying a pond in three months,’ claims one interviewee. Some tench farmers have tried to limit cormorants’ impact by stretching threads from side to side of the pond to block their entry (Fig. 6), though ‘they enter anyways, and when they are in, there is no way to drive them out.’ The problem peaks during winter (because less activity in the pond makes it more transparent and the lethargic state of the tenches makes them slower, which ease cormorants’ predation) and during spawning period (when tenches move close to the shores of the ponds and are exposed to predatory birds). The Louisiana crayfish is a globally invasive species [58]. It entered Piedmont in the 1980s, colonizing irrigation channels, rice fields, and *peschiere* alike (Fig. 7).^5^ Interviewees report that many ponds have been colonized by these crayfish, which heavily impact tench populations [61, 62]. They stress that even by emptying the *peschiera*, crayfish are not eradicated as they survive in the tunnels they dig on its bottom. Another invasive species from North America is the bullfrog, which frequents the *peschiere* eating tench eggs and fries, drastically reducing their number. Bullfrogs fill the ponds with voracious tadpoles that are a threat to the whole pond ecosystem by feeding from the benthic substrate. Some interviewees report that kilograms of these tadpoles can be caught with a single net pull in some ponds. A fourth invader is the swan mussel, an autochthonous bivalve of Italian rivers and lakes, where as a suspension feeder maintains the water transparent. Once introduced in the *peschiere*, this bivalve changes the pond ecosystem in an unfavorable way to tenches, which require organically rich, muddy waters. Interviewees report to have tried to empty some ponds and to leave them to dry to the August heat, but that this did not eradicate the mussels as they dig deep underground where they survive. The transfer of the bivalves into the ponds and from one pond to the other has occurred in connection with the transfer of fries and tenches between ponds; the mussels’ larvae hitched a ride on market and reciprocity networks.

The exchange of fries and tenches among households to replenish ponds, described as key to the social–ecological system, has been poisoned with pesticides and invasive species, which have become, in a manifestation of the Anthropocene in the ponds of the Pianalto, poison injected into once-nourishing relations [63]. Biological invasions are understood as a defining characteristic of the Anthropocene [64, 65]. Invading species can deeply affect ecosystems and are widely regarded as a threat to biodiversity [66]. In the *peschiere*, the Anthropocene manifests with global invasives such as the Louisiana crayfish and the nutrias, among others, which impact the functioning of the pond ecosystem and threaten the species that constitute it, including the tench. *Peschiere* become places where the particular and the universal, the local and the global, interact [67]. By linking the ponds’ ethnoecology and the tench ethnobiology to the Anthropocene, we situate the investigation in the context of the global environmental crisis and its manifestations [68]. This crisis is perhaps best captured by the concept of ‘Capitalocene’ as developed by Moore [69], with the focus on capitalism and capital circulation rather than on a flat ‘anthropos’ [70]. Capitalism is a way of organizing society that manifests globally with ecological destruction and social inequalities [71], and, as seen, makes itself felt in the ponds of the Poirino highlands through the conjoined effects of climate change, intensive agriculture, and invasive species.

### Sustainability and regional identity

Farming of the golden humped tench is part of the Italian finfish production from aquaculture [72], albeit a negligible one in quantitative terms. It is instead a relevant one as far as it concerns its quality of being integrated with livestock husbandry and connected to cropping through a key element of the Poirino highlands, artificial ponds called *peschiere*. The *peschiera* is a versatile feature, involved in building, milling, fish farming, agriculture, livestock husbandry, and water and landscape management, which can be framed within the social, cultural, and economic context of the highlands [36]. Ponds have been historically important in the livelihoods of rural inhabitants both for subsistence and for commercially [73]. Integrated pond aquaculture has developed in countless places into sophisticated systems of management and use, underpinned by a likewise sophisticated traditional knowledge [21–23]. Within the diversity of forms of integrated pond aquaculture [21, 24], a critical difference lies in the source of the nutrients applied (i.e., livestock or non-livestock based; from poultry, ruminants, or pigs). Perhaps, one of the defining features of tench farming in Poirino is that nutrients come from livestock manure that is released in the ponds directly by the animals watering or cooling there. The fertilization provided by livestock induces the production of food for the fish (i.e., algae, benthos, zooplankton) in the trophic web of the ponds’ ecosystems [74]. From this perspective, tench farming in the Pianalto is a little-known case of a traditionally designed integrated system of aquaculture, one revolving around artificial ponds called *peschier*e.

The system of Poirino highlands incorporates original elements and links (the house, furnace, pond, agriculture, livestock, tench), and as such it can inform not only the biocultural diversity of integrated aquaculture in ponds, but designs for similar systems for sustainability as well. Traditional integrated systems of aquaculture, their management and associated knowledge, can indeed be a model for the development of small-scale aquaculture in different settings [75, 76]. In a global context of increasing overall and per capita fish consumption [77], with marine resources rapidly declining [78] and booming intensive aquaculture of carnivorous species (and declining freshwater fish production in Italy [72]), the omnivorous tench and its extensive and integrated farming are an alternative sustainable option. Indeed, tench farming in ponds is in itself an exemplary case of sustainable integrated aquaculture, so much that it, as happens with ponds [79], has been used as a tool for environmental education [80].

Because these systems are the outcome of the coevolutionary process between human cultures and the territory they inhabit, they are unique in their elements and links, and as such they materialize in unique foods, carrying a distinct taste, ways of consumption, memories, rituals, and meanings [81–83]. These foods are being in many contexts valorized for their quality, through niche food markets and fairs, and as part of a specific gastronomic heritage [53, 84]. Local and traditional foods are object of attention in the quest toward sustainable food systems, and different trajectories of recovery and valorization are detectable [85]. We identified two trajectories of valorization taking place in the Poirino highlands, one more geared toward the social role of tench and tench farming and the other emerging as market-oriented for niche and typical products. The latter trajectory is, however, criticized by many interviewees for imposing a transformation of tench farming in connection with added feed, pond fencing, and the livestock ban from the pond, and a consequent disintegration of tench farming from livestock management.

Both trajectories contribute to the maintenance and reconfiguration of a collective identity rooted in a region and its constitutive elements [27, 86, 87]. Ponds and tench are such elements in the Poirino highlands; they represent a territory that is a sentimental, social, and symbolic product that underpins individual and collective identities. Ponds and tenches are core components of that affective bond between people and place underlying the notion of *topophilia* and maintained through *fields of care* that stem from people’s emotional attachment [28]. We argue that this identity is embodied in the highlands’ cultural landscape and is conveyed through the local gastronomic heritage as fried tenches and tenches *al carpione*. This identity relationship links the highlands’ community to the lived space and the species that inhabit it and is in continuous transformation vis-à-vis locals’ dynamic livelihoods. The golden humped tench emerges from this study as a keystone species in the social–ecological system of Poirino highlands.

With an ethnobiological and ethnoecological approach, this study contributes to the literature addressing social–ecological systems and their constitutive species [3, 4] and to the emerging fields of ethnoichthyology and aquatic ethnobiology [9, 10, 13, 14, 16]. The use of an ethnobiological approach to study traditional systems of integrated aquaculture is especially justified by the fact that these systems are knowledge-intensive and diverse [10, 21], and ethnobiological research on the topic deserves consideration. Given that the ponds as cultural landscapes are the result of sustained direct human interaction with ecosystems, *peschiere* and farms can also be understood as anthropogenic biomes, or anthromes [88, 89], i.e., ‘human systems, with natural ecosystems embedded within them’ [89] (p. 445). Anthromes encompass local subtle ecologies consisting of relations between people and place that ‘rely on diffuse causalities and micro-effects related to invisible or fleeting action’ [90] (p. 272). By understanding *peschiere* as anthromes, this study contributes to the literature addressing conservation and change in biodiversity and livelihoods in aquatic environments [88] and to the literature discussing the effects of global capitalism for aquatic anthromes [91]. In speaking of aquatic anthromes, global connections, environmental regulations, and the Anthropocene, this study eventually bridges ethnobiology with political ecology [20, 92].

## Conclusions

This study is an endeavor to discuss sustainable food production and regional identity as they relate to and emerge from traditional systems of integrated pond aquaculture. Using the farming of the golden humped tench of Poirino highlands as a case study, we have presented and discussed the ethnobiology of this local variety of tench and the ethnoecology of the ponds where tenches are bred, i.e., the *peschiere*. We tracked the history of the golden humped tench until the contemporary processes of valorization of the fish as a traditional and typical product, of its recognition as an element of regional identity, and of the emergence of new challenges that threaten its continuity.

The farming of the golden humped tench is a case of integrated fish farming and sustainable aquaculture. It is traditionally embedded in a peasant social–ecological system encompassing cropping and livestock husbandry, and revolving around the *peschiera*. The omnivorous tenches feed from the benthic substrate, which is renewed by the ponds’ fertilization by livestock watering in them. Such integrated system is informed by traditional ecological knowledge about the *peschiera* and its management, by ethnobiological knowledge about the fish and its life cycle, and by gastronomic knowledge on its processing, preparation, and consumption.

We have argued that traditional aquaculture systems and the knowledge that underpins them should be investigated and preserved in light of their role for sustainable food production. This study contributes to the literature on aquatic ethnobiology and ethnoichthyology by focusing on the golden humped tench and adds to the literature on traditional aquaculture by exploring a little-known case of integrated fish farming. By combining ethnoichthyology with an ethnoecological approach, this study can serve as model for future research in human–fish relationship in contemporary Europe, a field that is gaining interest within ethnobiology. The study finally contributes to the understanding of the key role of particular species in social–ecological systems and of the importance of these species for people’s livelihoods and culture.

## Data Availability

Please contact author for data requests.

## Footnotes

1 *Il carpione* refers to a processing method and to a way to marinate fish common across northwest Italy, whereby fish is fried and stored in a vinegar souse.

2 Tinca di Ceresole d’Alba—Presìdi Slow Food. (2020, November 18). Fondazione Slow Food. https://www.fondazioneslowfood.com/it/presidi-slow-food/tinca-di-ceresole-dalba/

3 European Commission 2008 Commission Regulation of 21 February 2008 registering certain names in the Register of protected designations of origin and protected geographical indications (Pane di Matera—PGI, Tinca Gobba Dorata del Pianalto di Poirino—PDO), 160/2008/EC Official Journal L 48 22 02 2008 27.

4 Associazione Produttori della Tinca Gobba Dorata del Pianalto di Poirino | Aree protette Po piemontese. (2021). Associazione Produttori della Tinca Gobba Dorata del Pianalto di Poirino. http://www.parcopopiemontese.it/dettaglio_azienda.php?id=12835

5 The tench had colonized the US a hundred years before the arrival of the Louisiana crayfish to the Poirino highlands. In 1922, an Italian emigrant brought some live tenches from Italy to California. The fish were bred in a pond and later other farm owners spread it extensively from pond to pond [59, 60].

## References

[1] Berkes F, Folke C, Colding J (2000). Linking social and ecological systems: management practices and social mechanisms for building resilience.

[2] Liu J, Dietz T, Carpenter SR, Folke C, Alberti M, Redman CL (2007). Coupled human and natural systems. AMBIO J Hum Environ.

[3] Garibaldi A, Turner N. Cultural keystone species: implications for ecological conservation and restoration. Ecol Soc. 2004;9(3).

[4] Volpato G, Di Nardo A (2017). The role of *Nucularia perrinii* Batt. (Chenopodiaceae) in the camel-based Sahrawi social-ecological system. J Ethnobiol Ethnomed.

[5] Rock M. Multi-species entanglements, anthropology, and environmental health justice. In: Routledge handbook of environmental anthropology. Routledge; 2016. p. 356–69.

[6] Jack J (2021). How bizarre, how bizarre, how bees are: domus and umwelt in the multispecies entanglements of humans and honeybees. Pathways.

[7] Gannon S (2017). Saving squawk? Animal and human entanglement at the edge of the lagoon. Environ Educ Res.

[8] Pieroni A, Pawera L, Shah GM, Albuquerque U, Nóbrega Alves R (2016). Gastronomic ethnobiology. Introduction to ethnobiology.

[9] García-Quijano CG, Pitchon A. Aquatic ethnobiology. Encycl Life Support Syst EOLSS. 2010.

[10] Svanberg I, Locker A (2020). Ethnoichthyology of freshwater fish in Europe: a review of vanishing traditional fisheries and their cultural significance in changing landscapes from the later medieval period with a focus on northern Europe. J Ethnobiol Ethnomed.

[11] Anderson EN (1967). The ethnoichthyology of the Hong Kong boat people.

[12] Morrill WT (1967). Ethnoicthyology of the Cha-Cha. Ethnology.

[13] Morales EMQ, Lepofsky D, Berkes F (2017). Ethnobiology and fisheries: learning from the past for the present. J Ethnobiol.

[14] Svanberg I (2015). Ræstur fiskur: air-dried fermented fish the Faroese way. J Ethnobiol Ethnomed.

[15] Mendoza JN, Mattalia G, Prūse B, Kochalski S, Ciriaco A, Pieroni A (2021). “Wild fish are a blessing”: changes in fishing practices and folk fish cuisine around Laguna Lake. North Philipp J Ethn Foods.

[16] Gauvreau AM, Lepofsky D, Rutherford M, Reid M. “Everything revolves around the herring” the Heiltsuk–herring relationship through time. Ecol Soc. 2017;22(2).

[17] Silvano RAM, MacCord PFL, Lima RV, Begossi A (2006). When does this fish spawn? Fishermen’s local knowledge of migration and reproduction of Brazilian coastal fishes. Environ Biol Fishes.

[18] Begossi A, Silvano RA (2008). Ecology and ethnoecology of dusky grouper [garoupa, *Epinephelus marginatus* (Lowe, 1834)] along the coast of Brazil. J Ethnobiol Ethnomed.

[19] Zapelini C, Giglio VJ, Carvalho RC, Bender MG, Gerhardinger LC (2017). Assessing fishing experts’ knowledge to improve conservation strategies for an endangered grouper in the Southwestern Atlantic. J Ethnobiol.

[20] Wolverton S, Nolan JM, Ahmed W (2014). Ethnobiology, political ecology, and conservation. J Ethnobiol.

[21] Prein M (2002). Integration of aquaculture into crop–animal systems in Asia. Agric Syst.

[22] Nakajima T, Hudson MJ, Uchiyama J, Makibayashi K, Zhang J (2019). Common carp aquaculture in Neolithic China dates back 8,000 years. Nat Ecol Evol.

[23] Brummett RE (1999). Integrated aquaculture in Subsaharan Africa. Environ Dev Sustain.

[24] Edwards P (1998). A systems approach for the promotion of integrated aquaculture. Aquac Econ Manag.

[25] Pohl J. Regional identity. In: Smelser NJ, Baltes PB, editors. International encyclopedia of the social & behavioral sciences [Internet]. Oxford: Pergamon; 2001. p. 12917–22. https://www.sciencedirect.com/science/article/pii/B0080430767024888.

[26] Paasi A (2003). Region and place: regional identity in question. Prog Hum Geogr.

[27] Häkli J, Paasi A. Geography, space and identity. In: Voices from the North. Routledge; 2018. p. 141–55.

[28] Tuan Y-F (1990). Topophilia: a study of environmental perception, attitudes, and values.

[29] Avlijaš S, Ricciardi A, Mandrak NE (2018). Eurasian tench (Tinca tinca): the next Great Lakes invader. Can J Fish Aquat Sci.

[30] Hesthagen T, Sandlund O (2007). Non-native freshwater fishes in Norway: history, consequences and perspectives. J Fish Biol.

[31] Alaş A, Altındağ A, Yılmaz M, Kırpık MA, Ak A (2010). Feeding habits of tench (Tinca tinca L., 1758) in Beyşehir Lake (Turkey). Turk J Fish Aquat Sci.

[32] González G, Maze R, Dominguez J, Pena J (2000). Trophic ecology of the tench, Tinca tinca, in two different habitats in North-West of Spain. Cybium.

[33] Perrow M, Jowitt A, Johnsonf S (1996). Factors affecting the habitat selection of tench in a shallow eutrophic lake. J Fish Biol.

[34] Veronesi N. Le peschiere del Pianalto di Poirino e la loro utilizzazione ittica. In: Studi geografici su Torino e il Piemonte. Edizione Giappichelli; 1954.

[35] Azzi L. Disciplinare di produzione della tinca gobba dorata del Pianalto di Poirino. Comune di Poirino (Torino); 2008.

[36] Pistarino A, Rota F (2008). Le, “Peschiere” di Ceresole d’Alba. Riv Piem St Nat.

[37] Julini M, Zoccarato I. Le tinche di Ceresole d’Alba. Terre Rosse Assoc Artist-Cult Roero Astisio Publ Bra CN Italy. 2000;31–55.

[38] Bernard HR (2017). Research methods in anthropology: qualitative and quantitative approaches.

[39] Parker C, Scott S, Geddes A. Snowball sampling. SAGE Research Methods Foundations; 2019.

[40] AAA. Code of ethics of the American Anthropological Association [Internet]. American Anthropological Association; 1998 [cited 2016 Jun 15]. http://www.aaanet.org/profdev/ethics/upload/ethicscode1998.pdf.

[41] Guest G, MacQueen KM, Namey EE (2011). Applied thematic analysis.

[42] Strauss A, Corbin J (1990). Basics of qualitative research.

[43] Lusso A (2008). Storia ed estetica del paesaggio. Tinche e peschiere a Ceresole d’Alba.

[44] Aplin G (2007). World heritage cultural landscapes. Int J Herit Stud.

[45] Jones M. The concept of cultural landscape: discourse and narratives. In: Landscape interfaces. Springer; 2003. p. 21–51.

[46] Domosh M, Smelser N, Bates P (2004). Cultural landscape in environmental Studies. International encyclopaedia of the social and behavioural sciences.

[47] Presti RL, Kohlmann K, Kersten P, Gasco L, Stasio LD (2010). Tinca Gobba Dorata del Pianalto di Poirino: genetic characterization by microsatellite markers. Ital J Anim Sci.

[48] Gasco L, Zoccarato I, Lussiana C, Azzi L, Julini M. Valorizzazione del territorio attraverso l’allevamento della tinca: il caso del Pianalto di Poirino (Piemonte). In: Editori MG; 2001. p. 109–14.

[49] Browne J, Lock M, Walker T, Egan M, Backholer K (2020). Effects of food policy actions on Indigenous Peoples’ nutrition-related outcomes: a systematic review. BMJ Glob Health.

[50] Strambi G (2010). I prodotti tradizionali e la politica di qualità dell’Unione europea. Riv Dirit Aliment.

[51] Mannia S (2018). Dop e slow food: formaggi siciliani fra politiche alimentari, strategie di mercato e retorica identitaria. Palaver.

[52] Lysaght P, Jönsson H, Burstedt A (2013). The return of traditional food.

[53] Bessière J (1998). Local development and heritage: traditional food and cuisine as tourist attractions in rural areas. Sociol Rural.

[54] Dees A, Roncarati A, Melotti P, Mordenti O. Prove di allevamento della varietà di tinca (Tinca tinca L.) finalizzate alla costituzione di uno stock di riproduttori ed alla produzione di giovanili in stagno. Parliamo Allev Altern E Valorizzazione Territ. 2004;175.

[55] Ciutti F, Beltrami ME, Confortini I, Cianfanelli S, Cappelletti C (2011). Non-indigenous invertebrates, fish and macrophytes in Lake Garda (Italy). J Limnol.

[56] Ciutti F, Flaim G, Beltrami M, Cappelletti C (2013). Non-indigenous fish fauna in Trentino lakes (Northern Italy). Quad ETP.

[57] Cowx IG (2015). Characterisation of inland fisheries in Europe. Fish Manag Ecol.

[58] Oficialdegui FJ, Sánchez MI, Clavero M (2020). One century away from home: how the red swamp crayfish took over the world. Rev Fish Biol Fish.

[59] Baughman J (1947). The tench in America. J Wildl Manag.

[60] Shapovalov L. The Tench in California. California Fish and Game. 2011.

[61] Savini D, Occhipinti-Ambrogi A (2008). Bad Moon Rising: il gambero rosso della Louisiana, una minaccia per gli ecosistemi acquatici della Lombardia. Mem Della Soc Ital Sci Nat E Mus Civ Storia Nat Milano.

[62] Delmastro G. Il gambero della Louisiana Procambarus clarkii (Girard 1852) in Piemonte: nuove osservazioni su distribuzione, biologia, impatto ed utilizzo (Crustacea: Decapoda: Cambaridae). Riv Piemontese Storia Nat. 2017;38:61–129.

[63] Van Dooren T (2010). Pain of extinction: the death of a vulture. Cult Stud Rev.

[64] Capinha C, Essl F, Seebens H, Moser D, Pereira HM (2015). The dispersal of alien species redefines biogeography in the Anthropocene. Science.

[65] Christoph K (2017). Plant invasions in the Anthropocene. Science.

[66] Simberloff D (2011). How common are invasion-induced ecosystem impacts?. Biol Invasions.

[67] Massey D. The conceptualization of place. Place World Places Cult Glob. 1995;45–85.

[68] Wyndham FS, Lepofsky D, Tiffany S (2011). Taking stock in ethnobiology: where do we come from? What are we? Where are we going?. J Ethnobiol.

[69] Moore JW (2017). The Capitalocene, part I: on the nature and origins of our ecological crisis. J Peasant Stud.

[70] Malm A, Hornborg A (2014). The geology of mankind? A critique of the Anthropocene narrative. Anthr Rev.

[71] Moore JW (2015). Capitalism in the web of life: ecology and the accumulation of capital.

[72] Parisi G, Terova G, Gasco L, Piccolo G, Roncarati A, Moretti VM (2014). Current status and future perspectives of Italian finfish aquaculture. Rev Fish Biol Fish.

[73] Locker A (2018). Freshwater fish in England: a social and cultural history of coarse fish from prehistory to the present day.

[74] Lin CK, Teichert-Coddington DR, Green BW, Veverica KL. Fertilization regimes. In: Dynamics of pond aquaculture. CRC Press; 2017. p. 73–107.

[75] Pullin R. Aquaculture, integrated resources management and the environment 1. In: Integrated fish farming. Taylor & Francis; 2020. p. 19–44.

[76] Prein M, Ahmed M (2000). Integration of aquaculture into smallholder farming systems for improved food security and household nutrition. Food Nutr Bull.

[77] FAO. The State of World Fisheries and Aquaculture 2020. Sustainability in action. Rome: FAO; 2020.

[78] Berkes F, Hughes TP, Steneck RS, Wilson JA, Bellwood DR, Crona B (2006). Globalization, roving bandits, and marine resources. Science.

[79] Kimmerer RW (2013). Braiding sweetgrass: indigenous wisdom, scientific knowledge and the teachings of plants.

[80] Faso RPFL, Gemio RV, García JCE, Ceballos-Zúñiga EG, Bueno C, Gallardo JM (2006). Tench, Tinca tinca (L.), fish farms as a tool for environmental education. Aquac Int.

[81] Abarca ME, Colby JR (2016). Food memories seasoning the narratives of our lives. Food Foodways.

[82] Holtzman JD (2006). Food and memory. Annu Rev Anthr.

[83] Franco FM, Bakar N (2020). Persistence of the salty-sweet nipah sugar in the popular foodways of Brunei Darussalam. J Ethnobiol.

[84] Fontefrancesco MF (2020). Food festivals and local development in Italy.

[85] Zocchi DM, Fontefrancesco MF, Corvo P, Pieroni A (2021). Recognising, safeguarding, and promoting food heritage: challenges and prospects for the future of sustainable food systems. Sustainability.

[86] Snow D. Collective identity and expressive forms [Internet]. UC Irvine: Center for the Study of Democracy; 2001 https://escholarship.org/uc/item/2zn1t7bj;jsessionid&.

[87] Volpato G, Howard P (2014). The material and cultural recovery of camels and camel husbandry among Sahrawi refugees of Western Sahara. Pastoralism.

[88] Martin LJ, Quinn JE, Ellis EC, Shaw MR, Dorning MA, Hallett LM (2014). Conservation opportunities across the world’s anthromes. Divers Distrib.

[89] Ellis EC, Ramankutty N (2008). Putting people in the map: anthropogenic biomes of the world. Front Ecol Environ.

[90] Wyndham FS (2009). Spheres of relations, lines of interaction: subtle ecologies of the Rarámuri landscape in northern Mexico. J Ethnobiol.

[91] Narchi NE, Cristiani BC (2015). Subtle tyranny: divergent constructions of nature and the erosion of traditional ecological knowledge in Xochimilco. Lat Am Perspect.

[92] Wolverton S, Nolan JM, Fry M. Political ecology and ethnobiology. In: Introduction to ethnobiology. Springer; 2016. p. 75–82.

